# The Influence of Etching Method on the Occurrence of Defect Levels in III-V and II-VI Materials

**DOI:** 10.3390/nano14191612

**Published:** 2024-10-09

**Authors:** Kinga Majkowycz, Krzysztof Murawski, Małgorzata Kopytko, Krzesimir Nowakowski-Szkudlarek, Marta Witkowska-Baran, Piotr Martyniuk

**Affiliations:** 1 Institute of Applied Physics, Military University of Technology, 2 Kaliskiego St., 00-908 Warsaw, Poland; krzysztof.murawski01@wat.edu.pl (K.M.); malgorzata.kopytko@wat.edu.pl (M.K.); piotr.martyniuk@wat.edu.pl (P.M.); 2 VIGO Photonics S.A., 129/133 Poznanska St., 05-850 Ozarow Mazowiecki, Poland; knszkudlarek@vigo.com.pl (K.N.-S.); mwbaran@vigo.com.pl (M.W.-B.)

**Keywords:** DLTS, MOCVD, MBE, MCT, T2SL, wet etching, ICP RIE, mixed etching

## Abstract

The influence of the etching method on the occurrence of defect levels in InAs/InAsSb type-II superlattice (T2SLs) and MCT photodiode is presented. For both analyzed detectors, the etching process was performed by two methods: wet chemical etching and dry etching using an ion beam (RIE—reactive ion etching). The deep-level transient spectroscopy (DLTS) method was used to determine the defect levels occurring in the analyzed structures. The obtained results indicate that the choice of etching method affects the occurrence of additional defect levels in the MCT material, but it has no significance for InAs/InAsSb T2SLs.

## 1. Introduction

The development of infrared (IR) detector technology is still being driven by its increasingly wide range of application possibilities. However, the fabrication/processing of high-quality MCT or InAs/InAsSb II-type superlattice (T2SLs) epitaxial layers for use in IR detectors requires knowledge and control of the many experimental factors that can cause material defects. The presence of defects in semiconductors deforms their band structure, resulting in the creation of additional levels in the band gap and energy bands that create trapping centers for carriers. As a result, the lifetime and mobility of carriers are shortened, affecting the parameters of optoelectronic devices. Each material is characterized by different defects. In the case of MCT, Hg vacancies are the main native defects. Additionally, dislocations [1,2,3], surface crater defects [4,5], stacking faults and twins [3,6], and pyramidal hillocks [1,7] may occur. For the InAs/InAsSb T2SLs, we distinguish native point defects (NPDs) such as In vacancies (V_In_), As vacancies (V_As_), or Sb vacancies (V_Sb_), as well as frequently occurring InAs (As_In_) or Sb_In_ (In_Sb_) interstitial defects [8,9]. Defect levels may be an inherent material parameter itself, or they may be introduced during technological processing. The choice of etching method depends on the adaptation of the device to specific applications. In semiconductor manufacturing, many etching techniques are used to create complex patterns and structures on material surfaces, with the primary methods being “dry” and “wet” etchings. Both of these methods have a number of advantages and disadvantages that may result in additional defect levels in the analyzed structure. Deep-level transient spectroscopy (DLTS) is a technique for the characterization of electrically active defects in semiconductors [10]. DLTS uses a semiconductor structure that contains a depletion region, usually a Schottky diode or p-n junction [11,12]. This method is based on the phenomenon of the capture and emission of charge carriers from the defect level and the related change in the capacity of the p-n junction [13].

## 2. Etching Methods

### 2.1. Wet Chemical Etching

One of the methods used for semiconductor structures’ processing is wet chemical etching. Wet etching is a material removal process that is not protected by masks. These masks are deposited and patterned onto the structure before the etching process using photolithography. For MCT digestion, a Br:HBr solution (1:100) diluted in deionized water (50:50:1 Br:HBr:H_2_O) is used. In the case of T2SL wet chemical etching, a solution of orthophosphoric acid–citric acid–hydrogen peroxide–water (molar ratio: 1:1:4:16) solution and 0.4 mol/dm^3^ hydrochloric acid water solution is used [14].

### 2.2. Dry Etching

In the dry etching process, the main role is played by physical processes (knocking out semiconductor atoms with high-energy gas particles) and chemical processes (reaction of free radicals with the etched material and then transition to the gas phase). In our research, the reactive ion etching (RIE) technique was used with inductively coupled plasma (ICP). The etching process was carried out in a mixture of working gases: SiCl_4_ and argon, which constituted the source of ions.

## 3. Measurement Technique

DLTS is the most powerful method used to characterize electrically active defects in semiconductors [8]. This method enables the detection of deep energy levels through the change in capacitance of a space charge region under the influence of a voltage applied to the p-n junction. A change in voltage results in a modification of the width of the depletion region, which in turn affects the junction capacitance. In addition, the capacitance changes due to the capture and emission of carriers from the trap levels. The capacitance versus time, *t*, is given by [15]:

(1) $$∆C\left(t\right)={\Delta C}_{0}e^{\frac{-t}{\tau }},$$

with $∆C\left(t\right)=C\left(t\right)-C_{\infty }$, where $C_{\infty }$ is the steady-state capacitance (t=∞) and τ is the time constant that is inversely proportional to the emission rate of the trap, $\tau =1/e_{n}$. The term$\;∆C_{0}$ represents the alteration in junction capacitance resulting from a change in voltage.

In the rate window (RW), i.e., between two times *t*_2_ and t_1_, the total capacitance change is measured:

(2) $$C\left(t_{2}\right)-C\left(t_{1}\right)=\Delta C_{0}\left[exp\left(\frac{-t_{2}}{\tau }\right)-exp\left(\frac{-t_{1}}{\tau }\right)\right],$$

during a gradual alteration in the temperature of the sample.

The Arrhenius plot from the emission rate equation could be given by:

(3) $$ln(T^{2}/e_{n})=ln\left(\frac{T^{2}}{\sigma_{n}\upsilon_{th}\frac{g_{0}}{g_{1}}N_{c}}\right)+\frac{E_{a}}{kT},$$

This makes it possible to determine the specific parameters that characterize the defect: activation energy at trap level $E_{a}$, and capture cross-section $\sigma_{n}$ [T—temperature, k—Boltzmann constant, $v_{th}$—thermal velocity, $N_{c}$—conduction band density of states and $\frac{g_{0}}{g_{1}}$—degeneracy coefficient ($g_{1}$—when traps are occupied, and $g_{0}$—when traps are empty)]. The level of the trap is measured in relation to the edge of the valence band (VB) and is expressed as follows: $E_{t}$: $E_{a}=E_{C}-E_{t}$ for electrons and $E_{a}=E_{V}+E_{t}$ for holes.

## 4. Experiment and Results

**Figure 1:**
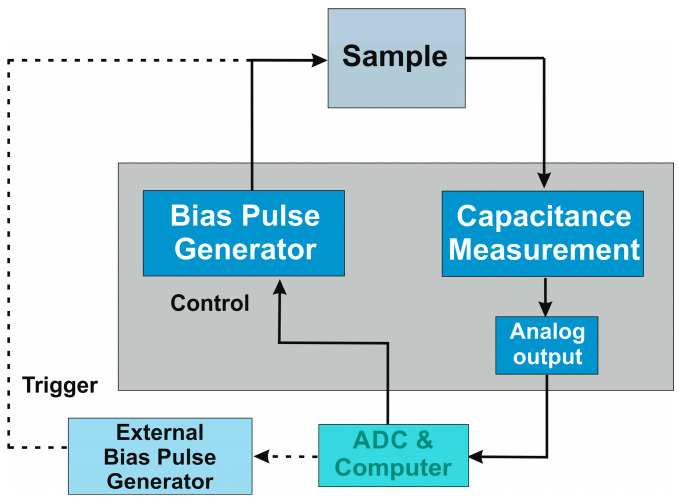
DLTS measurement system.

DLTS measurement for both detectors was performed on mesa-type structures with a surface area of 400 × 400 μm^2^. The single pixel was defined by standard optical photolithography and then by wet chemical and dry etchings. We have used a complete system of hardware and software from Semetrol (Chesterfield, VA, USA). The method uses a double “box-car” to define RW. DLTS measurements were performed in a closed-circuit helium cryostat, which allowed the temperature to be maintained in the range of 50–300 K. A diagram of the measuring system is shown in Figure 1.

**Figure 2:**
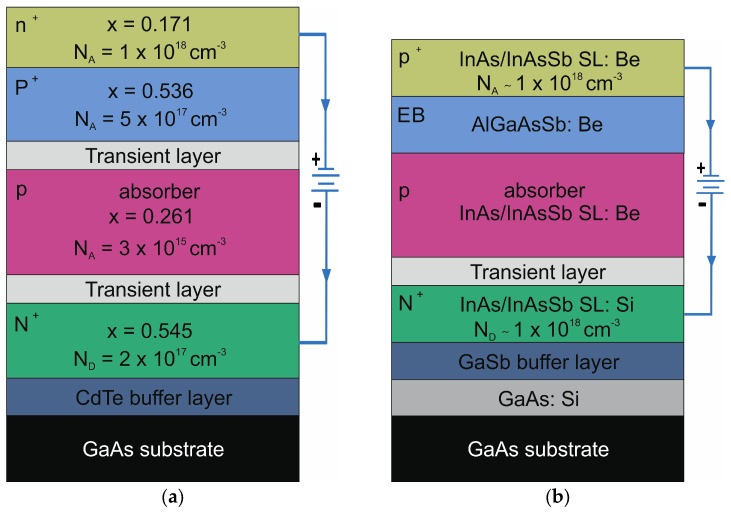
Structures for DLTS measurement: (**a**) MCT heterostructure; (**b**) InAs/InAsSb T2SLs.

For MCT, the conducted experiments are a continuation of the previously performed research described in refs. [16,17,18]. The medium-wave infrared (MWIR) MCT detector was fabricated with an N^+^/T/p/T/P^+^/n^+^ configuration by metal–organic chemical vapor deposition (MOCVD). A heavily In-doped N+ contact layer with a composition of x = 0.545 and a thickness of ~10 μm is located at the bottom of the structure. Next is the p-type As doped absorber with a composition of x = 0.261. The absorber is about 3.4 μm thick. Above the absorber is a wide band gap, heavily doped with As, with a 2 μm thick P^+^ layer with a composition of x = 0.536. On the top side of the whole structure, there is a 1 µm thick n+ layer with a lower composition than that of the absorber (x = 0.171) and a donor concentration of about 10^18^ cm^−3^. The MCT epilayer was grown on a 2″ epi-ready, semi-insulating (100) GaAs substrate after the CdTe buffer layer (see Figure 2a). The T2SLs InAs/InAsSb-based detector was fabricated in the p^+^Bppn^+^ configuration by molecular beam epitaxy (MBE) on a GaAs substrate. The p-type absorber was made of 578 × Period 4.39 nm (3.12 nm InAs/1.28 nm InAsSb, x_Sb_ = 0.34) T2SLs. Its architecture allows for work in the ~5.3 μm range at a temperature of 150 K. The wide band gap barriers surround the absorber on both sides. A 0.47 μm thick highly n-doped InAs/InAsSb T2SLs was used as the bottom contact N^+^ layer. Above the absorber layer is an electron-barrier (EB) made of a p-type AlGaAsSb. Next, there is a 0.1 μm thick highly p-doped InAs/InAsSb T2SLs, which provides electrical contact (see Figure 2b) [16]. For both structures, the top and bottom layers are covered with electrical contacts (Cr/Au).

### 4.1. Wet and Mixed Etching for MCT Structure

**Figure 3:**
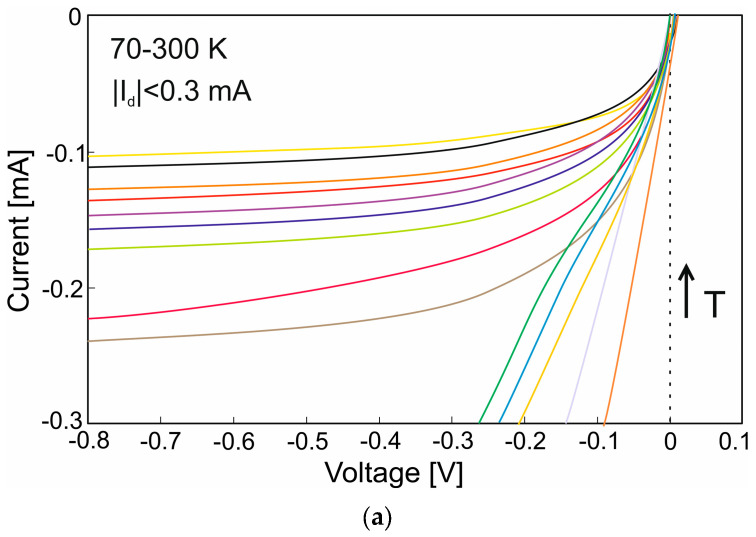

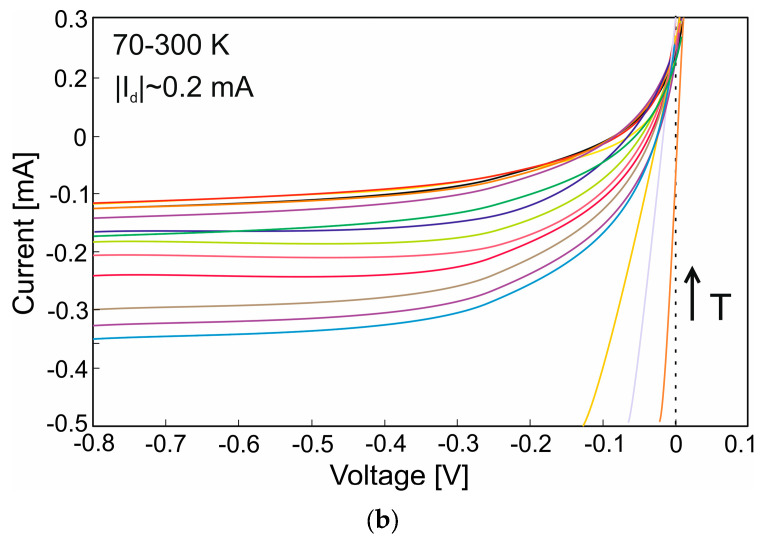
Current–voltage characteristics for MCT etched by (**a**) dry (ICP RIE), and (**b**) mixed (ICP RIE and wet) methods, (I_d_—dark current).

**Figure 4:**
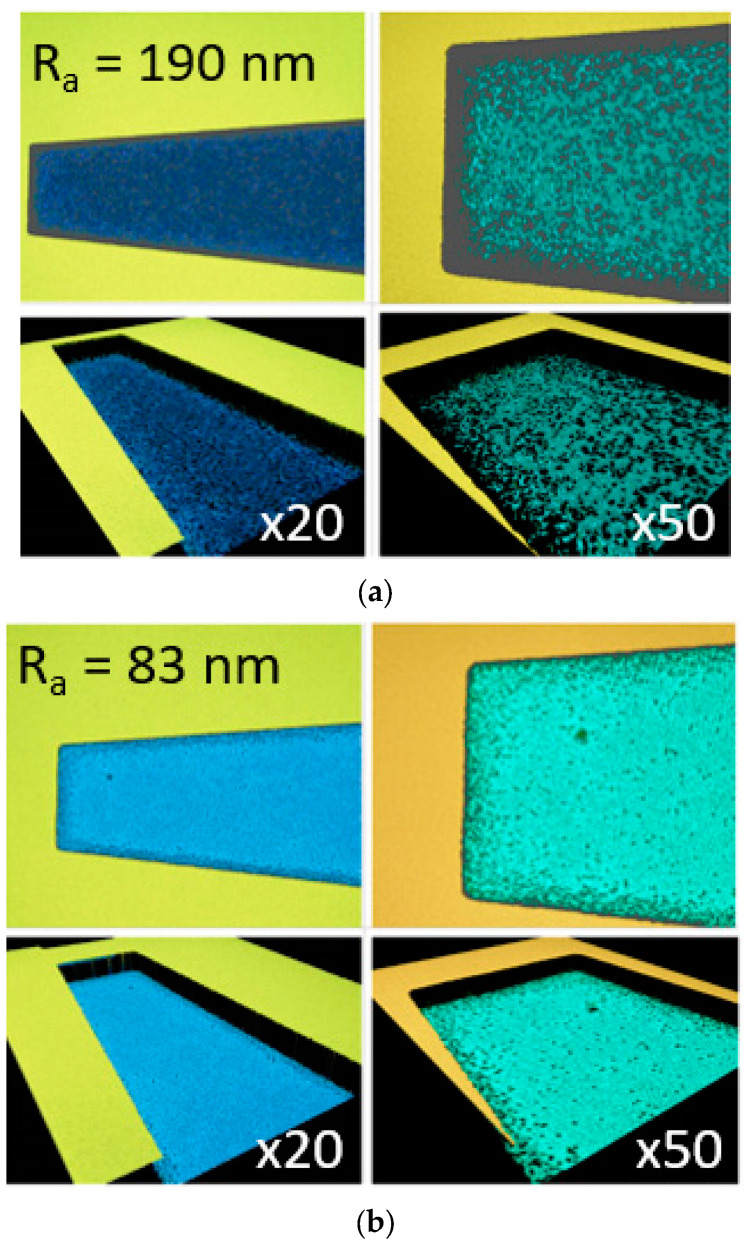
Surface roughness for MCT etched by (**a**) dry (ICP RIE) and (**b**) mixed (ICP RIE and wet) methods.

Figure 3 shows the current-voltage characteristics versus temperature (70–300 K) for MCT material etched using the dry [ICP RIE—(a)] and mixed [ICP RIE and wet—(b)] etching methods. The mixed etching technique involves etching the mesa-type structure using the ICP RIE and then lightly etching it in a diluted solution using the wet method. This is to “polish” the surface and remove oxides from the top of the layer. For the mixed etching method, the I-V characteristic has a similar shape for the entire temperature range. The detector exhibits dark currents of approximately 0.02 mA for reverse bias. Analyzing the surface roughness for both etching methods (see Figure 4), the R_a_ parameter is smaller for the mixed etching (83 nm) technique than for the ICP RIE method (190 nm) (where R_a_ is the arithmetic mean deviation from the mean line).

**Figure 5:**
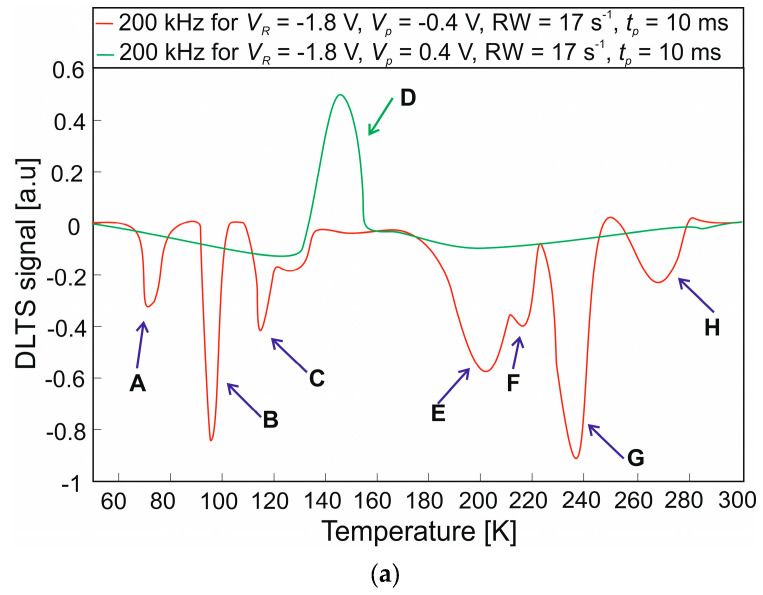

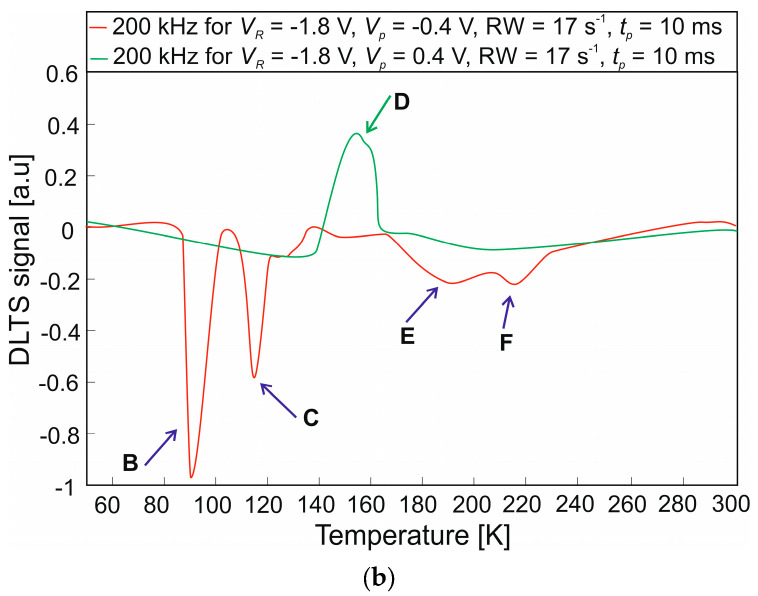
DLTS signal for MCT heterostructure: (**a**) wet etching; (**b**) mixed etching.

DLTS measurements were performed in the temperature range of 50–300 K for 200 kHz and tp = 10 ms filling pulse. The voltage applied to the device when measuring was set to *V_R_* = −1.8 V and the voltage applied to the filling traps (filling pulse) was set to a value of *V_p_* = −0.4 V and *V_p_* = 0.4 V. The DLTS signal is defined as *C*(*t*_2_)–*C*(*t*_1_) in our system, so that positive peaks correspond to majority-carrier traps, while the negative peaks come from the minority-carrier traps. Figure 5 shows the DLTS signals for the MCT heterostructure etched by the wet method (Figure 5a) and the mixed technique (Figure 5b). For wet etching, eight defect levels were obtained, designated A to H. One defect level (D) was observed at 150 K with a positive fill pulse (majority carriers). Three defect levels (A–C) were observed at low temperatures and four (E–H) at high temperatures (minority carriers). To isolate defects from individual layers, three different experiments were carried out on the same epitaxial layer, as described in [17]. Referring to the mentioned paper, the defect levels designated as B, C, D, G, and H should be considered. The trap parameters determined by Arrhenius plots (activation energies *E_a_* and capture cross-sections *σ*) are shown in Table 1. There are three defect levels designated as B, C, and D in the absorber layer [where *E_g_* for the absorber layer is 177 meV for 77 K and 240 meV for 300 K]. The peak B and C are from minority carriers (electron traps), while peak D is identified with majority carriers (hole traps). The calculated energy levels of B and C peaks are *E_C_* − 175 meV and *E_C_* − 147 meV, with the corresponding electron capture cross sections of 8.2 × 10^–13^ cm^2^ and 1.2 × 10^–16^ cm^2^. With a hole capture cross-section of 9.8 × 10^–12^ cm^2^, the estimated energy level for peak D is *E_V_* + 134 meV. The occurrence of G and H peaks, whose defect level energy is larger than the absorber energy gap, suggests that they probably come from the wide-gap P^+^ layer or are surface defects. For the mixed etching method, five defect levels (B, C, D, E, and F) were obtained. Four of them come from electron traps and one from a hole trap. Their activation energy and capture cross-section are similar to the values of defect levels obtained for wet etching (see Table 1). The mixed etching method allowed for the elimination of the peaks (G and H) attributed to surface defects.

### 4.2. Wet and Dry Etching for InAs/InAsSb T2SL

**Figure 6:**
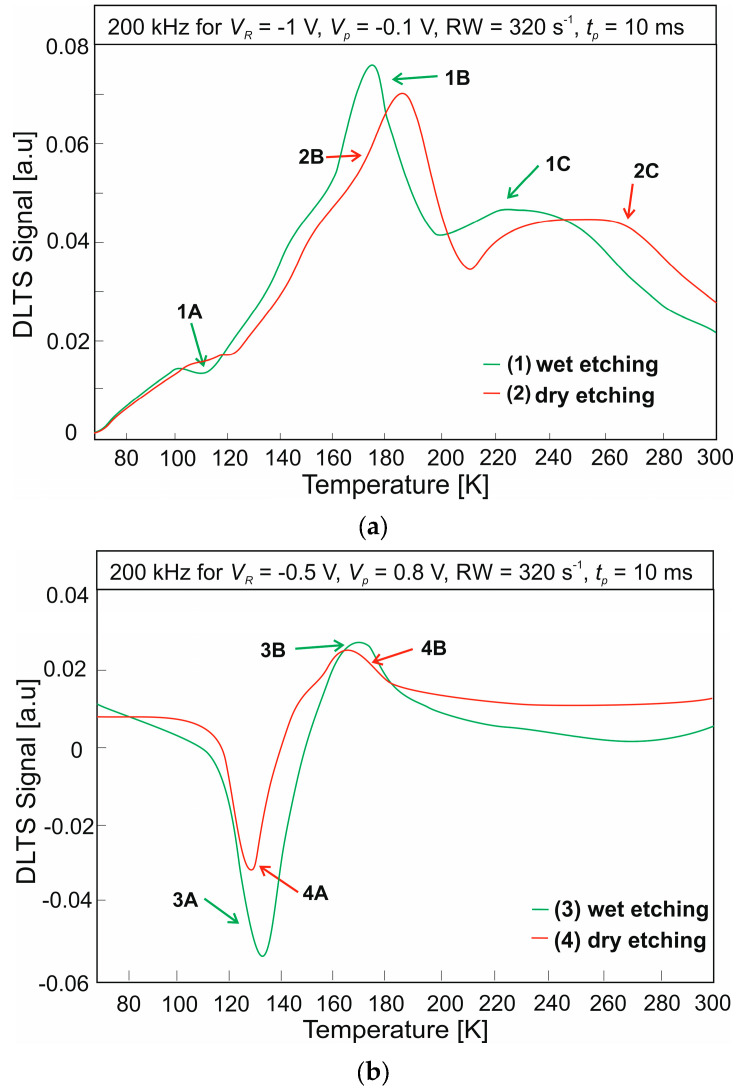
DLTS signal for wet and dry etched T2SLs InAs/InAsSb: (**a**) reverse bias; (**b**) forward bias.

Two different conditions were used for the DLTS measurements for T2SL. The photodiode was reverse-biased during the first measurement. The voltage applied to the device when measuring was set to *V_R_* = −1 V, and the filling pulse was set to *V_p_* = −0.1 V. The bias voltage was set to *V_R_* = −0.5 V in the second measurement and the fill pulse was set to *V_p_* = 0.8 V (forward-biased photodiode). Figure 6 shows the DLTS signals for the T2SL etched by the wet method and the ICP RIE. The measurements were performed for two different measurement conditions: reverse bias (Figure 6a) and forward bias (Figure 6b). For a wet-etched photodiode and reverse-biased, three defect levels were obtained. In the case of dry etching, two defect levels were obtained for the same voltage direction. The extracted defect levels come from the majority carriers. The measurement in the conduction direction allowed the identification of two defect levels stemming from the minority carriers for both etching methods. The trap parameters calculated by Arrhenius plots (activation energies, *E_a_* and capture cross-sections, *σ*) are shown in Table 2. The activation energies and the position of the peaks obtained during the DLTS measurement for both etching methods suggest that these are the same defect levels. Defect levels associated with peaks 1A, 1B (2B for dry etching), and 3A (4A for dry etching) are located in the absorber layer [$E_{g\;}$ = 245 meV (77 K) and $E_{g}$ = 196 meV (300 K)]. The calculated energy level of the 1B (2B) peak is $E_{V}\;$+ 220 meV ($E_{V\;}$+ 213 meV), with the corresponding hole capture cross sections of 1.2 × 10^–14^ cm^2^ (1.8 × 10^–14^ cm^2^). The estimated level of energy for the peak of 3A (4A) is as follows. $E_{C\;}$ − 86 meV ($E_{C\;}$ − 87 meV), with a hole capture cross-section of 6.8 × 10^–18^ cm^2^ (1.1 × 10^–19^ cm^2^).

## 5. Conclusions

This paper presents the results of the influence of the etching method on the occurrence of defect levels in MCT and T2SL InAs/InAsSb. The photodiodes were etched using two methods: dry (ICP RIE), and wet chemical and mixed. In the case of the MCT material, the use of the mixed etching method, where the sample was etched using the ICP RIE method and then subjected to wet etching, allowed for the elimination of surface defects. Additionally, the use of the mixed etching method improved the surface roughness, which affects the electrical contacts of the optoelectronic device. Three defect levels were identified in the active layer of the MWIR MCT heterostructure. Two of them were assigned to electron traps and one as a hole trap. For the photodiode made of InAs/InAsSb T2SLs, the etching method did not affect the occurrence of additional defect levels. The peaks obtained for the wet etching method are the same as those obtained in the case of dry etching. Their activation energies calculated using Arrhenius plots, the position of the signal peak, as well as the capture cross section are similar to each other. The extracted trap states are related to the inherent material properties and are not introduced during the processing steps of the detection device.

## Figures and Tables

**Table 1:** MCT trap parameters extracted by the DLTS measurements for wet and mixed etching.

*x_Cd_*	Peak	*T* [K]	*E_g_* [meV]	Wet Etching ∆*E_a_* [meV]	*E_t_* [meV]	Mixed Etching ∆*E_a_* [meV]	*E_t_* [meV]
0.261	B	100	185	$E_{C}$ − 175	10	$E_{C}$ − 171	14
0.261	C	120	190	$E_{C}$ − 147	43	$E_{C}$ − 152	38
0.261	D	150	198	$E_{V}$ + 134	134	$E_{V}$ + 154	154
0.261	E	200	211	$E_{C}$ − 194	17	$E_{C}$ − 202	9
0.536	G	240	618	$E_{C}$ − 336	281	-	-
0.536	H	280	617	$E_{C}$ − 351	266	-	-

**Table 2:** Trap parameters for T2SLs InAs/InAsSb extracted by the DLTS measurements for wet and dry etching.

Peak	*T* [K]	*E_g_* [meV]	∆*E_a_* [meV]	*E_t_* [meV]
Wet etching				
1A	110	240	$E_{C}$ − 108	132
1B	178	226	$E_{V}$ + 220	220
1C	230	215	$E_{V}$ + 589	589
3A	138	235	$E_{C}$ − 86	149
3B	170	228	$E_{V}$ + 291	291
Dry etching				
2B	182	226	$E_{V}$ + 213	213
2C	270	207	$E_{V}$ + 603	603
4A	130	238	$E_{C}$ − 87	151
4B	160	232	$E_{V}$ + 320	320

## Data Availability

Dataset available on request from the authors.
